# Vindication of Mr. Adams's Recommendation of Antim. Tart. in Egyptian Ophthalmia

**Published:** 1814-03

**Authors:** 


*For the Medical and Physical Journal.*

### *Vindication of Mr. Adams's Recommendation of Antim. Tart. in Egyptian Ophthalmia.*

THE truth of the old adage "none are so blind as those who will not see" is daily verified, and was especially so in a paper in a late Number of the Medical Journal.

Mr. W. M. T. of Hull, it appears, is unable to discriminate between the difference of the effects produced upon the constitution by "continual sickness" and by "continued sickness *and vomiting*," although Mr. Adams, subsequent to the appearance of a paper on the subject by Mr. Fielding, has given such a clear explanation of the different effects induced by the two modes of practice, as not only to have silenced Mr. Fielding in any further prosecution of his claims of priority, but also to have called forth from a gentleman of Clifton an excellent paper (in the third Number of the 30th volume, page 199, of the Med. and Phys. Journal) entitled *Distinction between Nausea and Vomiting*, in which the author says, "If the same effect be brought about by nausea as by active vomiting, it must be the product of causes diametrically opposite to each other." The correctness of his detail of the effects produced on the constitution by nausea, must be acknowledged by every candid and intelligent professional reader. He clearly shows that the animal and vital powers are much depressed during its action, even in the most athletic patient, who in consequence "becomes deprived of all muscular energy, occasioning the highest degree of prostration of strength and listlessness," while, by increasing the nauseating matter, he adds, "instead of increasing the nausea, we excite vomiting, and effects very opposite to those I have been describing are produced; for now, instead of a relaxation of the muscles, instead of a death-like listlessness, nature is roused to make a powerful effort to get rid of the offending matter; the muscles, not only of respiration, but also of the extremities, are thrown into strong contractions, and the heart and arteries propel their contents onward to the ultimate branches with increased velocity; so that it frequently happens, during violent vomiting, the delicate vessels of the conjunctiva covering the sclerotic coat are ruptured, and blood consequently extravasated into the cells of the reticular membrane, constituting what is called a blood-shot eye. The pulse, instead of being so quick and feeble as scarcely to be felt or counted, as we find it to be during nausea, now bounds under the finger; and the face, partly owing to the pressure of the contracted contracted muscles on the veins hindering the return of the blood through them, and partly to the increase of circulation, is suffused with a dark crimson hue, and apparently swollen, instead of being flaccid and deathly pale." This description being given in more animated language than that in Mr. Adams's preceding paper, (whose reasoning, by the bye, is equally correct,) I have chosen it for quotation, and as a proof that where there is a disposition to detract from another's merit, the clearest descriptions may be contorted, as well as facts mis-stated. I hope I shall not be considered to treat Mr. Fielding, and his fellow townsman Mr. W. M. T. with too much severity by these remarks; but I really think them justly called forth by the unfair attempts made by the one to deprive Mr. Adams of the merit of a very valuable discovery, in which, having failed, the other endeavours to prove it inefficacious, and in support of his assertion brings forward a case in which the disease itself, as well as the nature of his practice, *totally differ* from that recommended by Mr. Adams.

But let us examine the paper of Mr. W. M. T. by which it will appear that he has *mis-stated* some of Mr. Adams's assertions, and also *wholly mistaken* his mode of conducting the practice. He sets out indeed at the very commencement with a mistake of no small moment, namely, that Mr. Adams had recommended the use of the antim. tartar. for the cure of *acute ophthalmia.* Now that gentleman, in his two communications which have appeared in the Medical Journal, speaks only of having tried it in the *purulent inflammation of the conjunctiva, known under the appellation of the Egyptian ophthalmia*, which Mr. W. M. T. ought to have known is of a specific nature, and very different in its character and progress from simple acute inflammation, which I presume he means by "acute ophthalmia." That I am not incorrect in this presumption is, I think, clear, from the description of a case which Mr. W. M. T. adduces in support of his induction, in which are absent all the symptoms which characterize the *Egyptian ophthalmia*, when it exists in any degree of violence, namely, a copious puriform discharge from the conjunctiva, a great degree of tumefaction of the palpebræ, and an excessive vascularity and thickening of the conjunctiva, folds of which not unfrequently envelope the transparent cornea so as almost to obscure it from view, constituting what in technical language is called the chemosis. If Mr. W. M. T. had had any practical experience in the treatment of this disease, or had even perused any of the publications upon the subject, (that excepted by Mr. Ware, at which period it is impossible he could have ever seen the

1 ophthalmia ophthalmia in question in the virulent state in which it has existed in the army,) he would have known that "four ounces of blood from the temple and ten from the arm" would have done very little towards "affording the speedy recovery and preservation of sight" ascribed to it by him in the case he details. When he is therefore informed that it is frequently necessary to take from 60 to 80 and even 100 ounces of blood or upwards from the patient, to save the eyes from total destruction, in bad cases of Egyptian ophthalmia, he cannot fail to comprehend how totally different was the disease he had to treat.

It is next stated, in the paper from Hull, as Mr. Adams's opinion, "that the antim. tartar. alone, given in doses sufficient to keep up *continual sickness*, and continued for a sufficient length of time, was capable of subduing the inflammation *in the most violent cases*." If Mr. W. M. T. has been favored with any private communications by Mr. Adams on the subject, I cannot contradict his assertions; but as he does not give the slightest intimation in his paper to this effect, and judging Mr. A.'s opinions only as they are expressed in his two papers inserted in the Medical Journal, I must beg leave to say, that in these particulars he is equally incorrect as in the former. Mr. Adams, in his first paper on the ophthalmia, inserted in the 170th Number of this Journal, page 303, after mentioning that the mode of treatment recommended by a celebrated oculist had been ineffectually tried by himself and his son, for the space of two years, upon a large number of patients in St. Pancras Work-house, where he first tried his practice, and with uniform success, says, "The process was simply to give such a quantity of antim. tartar. as would keep up constant sickness *and vomiting* for eight or ten hours," &c. In Mr. Adams's second paper, written in reply to that of Mr. Fielding, he gives more detailed instructions for exhibiting the emetic tartar, which I will quote in his own words:—"To produce nausea, a quarter, or at most the third, of a grain of the antim. tartar is exhibited for a dose once, in three or four hours, to an adult; whereas in my practice I should direct *two grains* to be given at the first, and half that quantity to be repeated *every half hour*, until *full vomiting* is produced, which is to be kept up for the stated time, by repeating the dose at longer intervals." Now in the case related by Mr. W. M. T., it is true he gave a full grain of antim. tartar, instead of a fourth or a third part of that quantity, and that too with the addition of nitre, which, he says, produced the desired effect of keeping the patient in a state of "continual sickness" (alias nausea); but he never once, in any part of his paper, mentions the word

*vomiting*,

*vomiting*, which is insisted upon by Mr. Adams as indispensably necessary (that too in a violent degree) for the cure of the Egyptian ophthalmia, according to his mode of treatment. The *opposite effects* produced by sickness and by violent vomiting have been fully shown; and out of regard to the veracity of the anonymous writer in question, I am willing to suppose he has not perused Mr. Adams's papers himself, but taken them on hearsay; otherwise a more wilful perversion of the intentions of the inventor of a new practice has never yet been attempted.

Another deviation of most essential importance, in the Hull case, from Mr. Adams's practice, is, in the period of first exhibiting the antim. tartar. Mr. A., at the conclusion of his first paper on ophthalmia, says, *in italics*, that the emetic practice should be adopted "*as soon after the commencement of the disease as possible*, as the ulcerative process frequently begins in ten or twelve *hours* (which, by a note in his second paper, it appears was misprinted *days)* after the accession of inflammation, and, it is evident, the remedy, *when that stage of the disease has begun*, must be ineffectual;" whereas in the case narrated by Mr. W. M. T., the inflammation, he asserts, was of the most violent kind, pre-existing *three days* before the patient sent for advice, when the corneæ were found to be "dull and milky, and one of them *beginning* to *ulcerate*." Every medical practitioner is fully aware how much the efficacy of an emetic, in cutting short any acute affection, is dependant on its early administration: they also know that the cold affusion loses its power of immediately arresting the further progress of typhus, when applied later than the period named by the late eminent physician who reduced its application to the rules for its use which are now universally admitted. Would it therefore be surprising were Mr. Adams's practice to fail (even supposing it applied to the very disease in which he recommends its adoption) if administered *two days and a half* later than the period distinctly marked by Mr. A., beyond which it would frequently, he says, "be highly injurious?"

The author of the article from Hull, in his concluding sentence (in which by the way there is such a curious jumble of persons and tenses as to render it nearly unintelligible), says, "The object of the writer is not to condemn the use of emetic tartar in ophthalmia, but to express his opinion, grounded on observation, that it ought not to be relied on as a principal remedy; and from the manner in which Mr. Adams spoke of it, many, no doubt, would be led to place entire confidence in it, without ever thinking of any other until it was too late." Mr. Adams certainly does speak of the the efficacy of his practice with great confidence, when he says, "The event (of the emetic practice) has most fully answered these expectations, *as in no one instance* in which I have known the remedy employed agreeably to the rules just laid down, has it failed of success." In support of this assertion, *thirteen* cases are also mentioned, given by Mr. Lewis (one of the surgeons of St. Pancras Work-house) in a public document; also Mr. Adams announces that that gentleman has since successfully pursued the practice on other public as well as private patients. This fully warranted Mr. A. in "hastening to lay the facts before the public, with the wish of inducing its extensive adoption" in the *Egyptian ophthalmia*, but evidently not in a species of ophthalmia totally different from that most-destructive disease.

*Liverpool,* *An Admirer of Truth and Justice.*

*Jan.* 15, 1814.

